# Psychiatric hospital reform in low- and middle-income countries: a systematic review of literature

**DOI:** 10.1007/s00127-021-02075-z

**Published:** 2021-04-21

**Authors:** Tasneem Raja, Helena Tuomainen, Jason Madan, Dipesh Mistry, Sanjeev Jain, Kamala Easwaran, Swaran P. Singh

**Affiliations:** 1 Tata Trusts, World Trade Center, Cuffe Parade, Mumbai, 400005 India; 2 Mental Health and Wellbeing, Warwick Medical School, University of Warwick, Coventry, England; 3 Centre for Health Economics, Warwick Medical School, University of Warwick, Coventry, England; 4 Warwick Clinical Trials Unit, University of Warwick, Coventry, England; 5 Molecular Genetics Laboratory, Department of Psychiatry, National Institute of Mental Health and Neurosciences, Hosur Road, Bangalore, 560029 India; 6 Founder Sumunum Foundation, Chennai, India; 7 Centre for Mental Health and Wellbeing Research, University of Warwick, Coventry, England

**Keywords:** Psychiatric hospitals, Low- and middle-income countries, Transforming psychiatric hospitals, Reform of mental hospitals

## Abstract

**Purpose:**

Psychiatric hospitals or mental asylums grew across the world in the colonial era. Despite concerns over quality of care and human rights violations, these hospitals continue to provide the majority of mental health care in most low- and middle-income countries (LMICs). We sought to review the evidence of reform of mental hospitals and associated patient outcomes.

**Methods:**

We adopted an integrative review methodology by including experimental and non-experimental research. The review protocol was registered on PROSPERO (CRD42019130399). A range of databases and systematic hand searches were conducted by two independent reviewers. Research conducted between 1980 and May 2019, that focused on any aspect of reform in mental hospitals for adults (age 18 and upwards) with severe mental illness and published in English, were considered.

**Results:**

16 studies were included in the review. 12 studies met inclusion criteria, and four additional reports emerged from the hand search. Studies covered—India, China, South Africa, Grenada, Georgia, Sri Lanka, Argentina and Brazil. Key findings emphasise the role of judicial intervention as a critical trigger of reform. Structural reform composed of optimisation of resources and renovations of colonial structures to cater to diverse patient needs. Process reforms include changes in medical management, admission processes and a move from closed to open wards. Staff engagement and capacity building have also been used as a modality of reform in mental hospital settings.

**Conclusion:**

There is some documentation of reform in psychiatric hospitals. However, poor methodological quality and variation in approach and outcomes measured, make it challenging to extrapolate specific findings on process or outcomes of reform. Despite being integral service providers, psychiatric hospitals still do not adopt patient centric, recovery-oriented processes. Hence, there is an urgent need to generate robust evidence on psychiatric reform and its effect on patient outcomes.

## Introduction

Mental asylums are a hallmark of the globalization of psychiatry. Established globally during the colonial period, they continue to provide care for the severely mentally ill. They account for a majority of mental health care available in Low- and Middle-Income Countries (LMICs). Mental hospitals consume a bulk of the financial resources allocated for mental health. Despite the high admission rates into psychiatric care settings (99.1 per 100,000), the number of hospital beds per 100,000 is only 11.3, with over 18% of bed capacity being occupied by individuals with long-term care needs. With only nine mental health professionals per 100,000 people, the burden of caregiving is high [1]. Care provided in mental hospitals is shaped by a range of factors including legal reforms of the late twentieth and early twenty-first century [1–3].

Psychiatric hospitals remained an area of interest to the scientific community for a large part of the twentieth century; however, the interest sharply declined over the last three decades [4, 5]. Further, the emerging field of global mental health prioritized research on community mental health [6] particularly on common mental disorders and depression while neglecting mental hospitals. Hospitals and long-stay institutions do not feature in the top 25 Grand Challenges in Global Mental Health [7, 8]. The fight to improve the appalling conditions and reduce incidence of abuse in these hospitals has been left to media, non-governmental organizations and human rights commissions [9–13].There is a disconnect between this community-based focus of global mental health and the plight of the severely mentally ill who are often behind closed walls of institutions. Mental hospitals continue to remain an important provider of care and there is an urgent need to reform practices to improve quality of care and reclaim dignity for service users. LMICs need pragmatic and evidence-based approaches to psychiatric hospital reform, where there is a balance and optimisation of resources spent on community care vis-a-vis expenditure on psychiatric institutions [2].

## Objective

Given this backdrop, a systematic review of literature synthesizing research on psychiatric hospital reform, particularly in LMICs is essential to frame stronger, more appropriate reform programmes. The review aimed to understand the process and outcome of psychiatric hospital reform in LMICs by:

- Distilling evidence and scientific literature around mental hospital reform in LMICs and documenting process and outcome of reform.
- Understanding the impact of structural and process reform of psychiatric hospitals on patient outcomes in LMICs.
- Identifying gaps in current evidence and research with regard to the reform of psychiatric institutions in LMIC country settings.

## Methods

We adopted an integrative review methodology for this study. It includes the four steps of a systematic process i.e. search, appraisal, synthesis and analysis. It allows for inclusion of both experimental and non-experimental research. The review protocol was registered on PROSPERO CRD42019130399. We followed the Preferred Reporting Items for Systematic Reviews and Meta-Analyses (PRISMA) Statement and Consolidated Standards of Reporting Trials (CONSORT) Statement [14]. This review was undertaken as part of the Structured Individualised inTervention and Recovery (SITAR) study, which is embedded in a larger programme on psychiatric hospital reform called Udaan. Udaan is a partnership of Tata Trusts with government of Maharashtra, formalised through a Memorandum of Understanding, to develop the Regional Mental Hospital Nagpur (RMHN) as a centre of excellence through systematic reform of the hospital. Udaan (which in Hindi mean to ‘soar’) comprises four key reform elements: structural (refurbishing old colonial infrastructure to meet current service user needs), process (standardising clinical and non-clinical processes of the hospital), capacity building (standard training for different levels of hospital staff) and introduction of individual need-based, recovery-oriented, service package for patients [15]. Udaan defines psychiatric hospital reform as a care transformation process across the four domains of structural reform, process reform capacity building of staff and an individual patient services package. We have used this framework to operationally define reform for the purpose of this review. The SITAR study embedded within the Udaan program is a two-arm pragmatic randomised control trial which tests if an individual patient service package improves outcomes amongst long-stay in-patients in comparison to larger psychiatric hospital reform [15].

### Eligibility criteria

The review sought to identify papers that studied mental hospitals or similar care facilities in the 137 LMICs. Research conducted between 1980 and May 2019, that focused on any aspect of reform for adults (age 18 and upwards) with severe mental illness and published in English were considered. In addition, we did a citation search of all included publications. Studies excluded were: non-English publications, studies from high income countries, interventions in general hospital and community settings for adolescents/children (below age 18), and those that studied non-Severe Mental Disorders (SMDs). Studies published before 1980 were also excluded. The detailed PICOs for the review are in Table 1, below.

**Table 1:** PICOS for the systematic literature review

Population	People living in an institute Mental hospitals/psychiatric hospitals/asylums/psychiatric institutions in Low- and Middle-Income Country (LMICs) as defined by the World Bank
Intervention	Intervention in the institutional setting Transition/reform/change/modernization/improvement/de-institutionalization
Comparator	People who have not received the intervention or to the setting prior to the intervention A comparator is not necessary
Outcomes	Change in patient level indicators—symptoms, functionality, disability, social interaction, quality of life (any relevent measure/scale). Process indicators, such as length of stay and number of admission episodes
Study design	Randomized and non-randomized study designs (all publications) From 1980 till date

### Search strategy and data sources

A two-pronged search strategy was used (a) database search and (b) hand searching to identify relevant studies.

We searched five databases: Medline, PsycINFO, Web of Science, Scopus and Cochrane using the key words and combinations reflected in Table 2 below. We used a country specific search since the combination of key words for LMICs does not appear readily on databases. We conducted the search between Nov 2019 and February 2020 for all the databases. For the hand search, we examined the reference lists of all identified studies.

**Table 2:** Search strategy and syntax

Search terms	Resources
**Population**	
Adults	
**Setting**	
Mental hospital	
Psychiatric hospital	
Mental asylums	**Bibliographic and journal databases**
Psychiatric institutions	APA PsychINFO
	PubMed/medline
**Intervention**	Cochrane reviews
Reform	Web of science
Change	Scopus
Modernisation	
Improvement	
Deinstitutionalisation	
**Location**	
Low and middle-income countries (lower, mid, and upper mid income) as defined by the World Bank—137 countries	

### Data extraction and quality assessment

The first author (TR) ran the primary search, assessed eligibility criteria for all retrieved papers and assessed the quality of all included studies. The first author also extracted data for all included studies with 100% of the sample being extracted independently by another author (KE). RATS (relevance, appropriateness, transparency and soundness) qualitative research review guidelines were used for the quality assessment of the seven qualitative studies. The RATS scale comprises 25 questions that assess the relevance of the research question, appropriateness of the methods used, transparency of the study and methods and soundness of the approach used for interpretation of findings. For the purpose of this review, each question on the RATS scale was assigned a binary value (yes—1 point and no with 0 points) to effectively make a judgement on the quality of the included qualitative research papers. This approach was drawn from a previous systematic review using multiple types of studies [16, 17]. The Effective Public Health Practice Project (EPHPP) [18] was used to assess the quality of the five quantitative studies included in the review. The four reports included from the citation search of included studies were not assessed for quality since they are reports, three of them are country reports and one is a programme report.

Data were extracted and tabulated independently by two authors (TR and KE) for all papers meeting the eligibility criteria. The data extraction tool was developed by the first author (TR) and was modeled on the data extraction templates for RCTs and non-RCTs [19]. The tool comprised the following categories: general information (title of the study, study authors, type of study, journal of publication, year of publication, country of study), intervention setting (type of facility, study period, number of patients in the study, length of stay along with the admission and discharge process), costing details (annual budget of the institution), reform components (triggers for reform, elements of reform and cost of reform), and outcomes (patient data on clinical, social and functional outcomes).

## Results

**Fig. 1:**
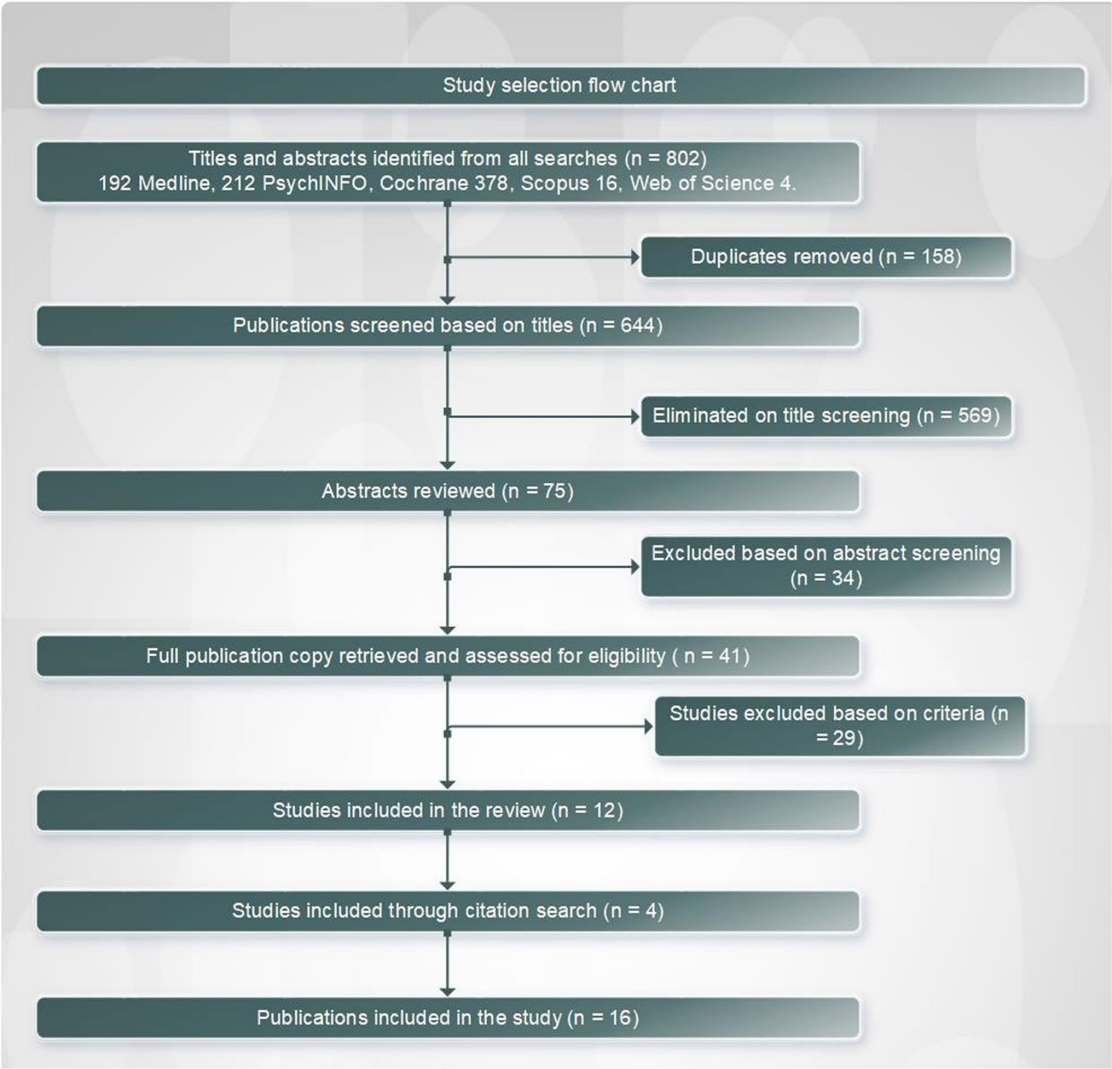
Study selection flow chart

802 studies were identified through the database search of which, following exclusion of duplicates, abstract reviews, and full-text reviews 12 studies met the inclusion criteria. The hand search yielded four additional reports (Fig. 1). Of the 16 studies included in this review, seven were varied qualitative studies including, two case studies, one personal reflection, one ethnographic study, one observational study and one historical study. Five of the 16 included articles and reports were quantitative studies. Of these, two were randomised controlled trials, one was a non-randomised control trial, one case–control study and one quasi-experimental study. The four publications found through citation search included three country-level reports and one programme report. Quality assessment indicated high variability with nine of the 12 assessed studies as weak, two as moderate and one as high quality.

### Data synthesis

A preliminary synthesis was developed using tabulation. Data were directly reported and cumulated where possible for quantitative variables. For qualitative data, emergent themes were drawn out and a vote count was undertaken to identify the frequency with which themes appeared. The studies covered a publishing period from 1994 to 2017 and represented eight countries: India [19–23], China [24–26], South Africa [27, 28], Brazil [29, 30], Argentina [31], Grenada [32], Georgia [33] and Sri Lanka [34]. All of the studies were based in state-run psychiatric hospitals. A total of 112 hospitals were covered through these studies, out of which, there were 60 unique hospital settings (studies in India were done in the same hospital). The number of hospitals covered per country ranged from 47 in India to one each in Grenada and Sri Lanka, [19–24, 26–36]. A high variability was found in the number of patients/number of beds with some studies covering as low as 10 patients [27] and the highest being 237 from India [22] Three qualitative studies do not mention any numbers [19, 20, 33]. Since there is high variability in the numbers reported, no further analysis was undertaken Characteristics of all included studies are reported in Table 3 below.

**Table 3:** Charecteristics of studies included in the systematic review

No	Quality rating	Full citation	Country	Method	Number of hospitals	Study participants	Number of patients/beds in hospital or unit	Duration of stay	Quality rating
1	EPHPP (score 3) weak	Uys, L., Mhlaluka, N. and Piper, S. (1996) An evaluation of the effect of programme changes in an acute psychiatric unit. *Curationis*, 19 (3): 21–27	South Africa	Quantitative study- quasi-experimental study	1	34	520 female admissions unit	–	EPHPP- (3) weak
2	RATS (score 5/25) weak	Varma, S. (2016) Disappearing the asylum: modernizing psychiatry and generating manpower in India. *Transcultural Psychiatry*, 53 (6): 783–803	India (Institute for Mental Health and Neurosciences- Kashmir)	Qualitative study- ethnographic study	1	–	–	–	RATS 5/25
3	RATS (score 3) weak	Murthy, P., Isaac, M. and Dabholkar, H. (2017) Mental hospitals in India in the twenty-first century: transformation and relevance. *Epidemiology and Psychiatric Sciences*, 26 (1): 10–15	India (RMH Pune, LGBRIMH-Assam, Hospital for mental health—Gujarat)	Qualitative-report	3	–	–	–	RATS 3/25
4	EPHPP (score 3) weak	Krüger, C. and Lewis, C. (2011) Patient and social work factors related to successful placement of long-term psychiatric in-patients from a specialist psychiatric hospital in South Africa. *African Journal of Psychiatry*, 14 (2):	South Africa (Weskoppies Hospital—10 wards accommodating long-stay patients)	Quantitative cross-sectional descriptive study	1	271	1067	12.78 years	EPHPP (3) weak
5	RATS (score 17/25) moderate	Bandeira, P. M., Haddad P. Souza, C., da Silva Guimarães, J. C., de Almeida Filho, A. J. and de Almeida Peres, M. A. (2015) Psychiatric nursing in integrated wards accommodating both female and male patients: a historic pioneering reform initiative implemented by the Institute of Psychiatry, a Unit of the Federal University of Rio De Janeiro, Brazil. *Issues in Mental Health Nursing*, 36 (10): 791–798	Brazil (Institute of psychiatry- Federal University- Rio de janeiro	Qualitative study- historical social study (thematic oral history technique	1	Four nurses and three nursing technicians	Two wards of 50 beds each		RATS 17/25
6	RATS (score 3/25) weak	Makhashvili, N. and van Voren, R. (2013) Balancing community and hospital care: a case study of reforming mental health services in Georgia. *PLoS Med*, 10 (1): e1001366	Georgia	Qualitative-case study	6		Average of 1000 beds each	–	RATS 3/25
7	RATS (score 13/25) weak	Jin, D. and Li, G. (1994) The role of human rights and personal dignity in the rehabilitation of chronic psychiatric patients: a rural therapeutic community in Yanbian, Jilin. *The British Journal of Psychiatry*, 165 (S24): 121–127	China (Yanbian community psychiatric hospital- branch)	Observational study, no comparator/control group	1	81 patients with schizophrenia	120 total patients	14.2 years	RATS 13/25
8	EPHPP (score 3) weak	Fan, Z., Huang, J., Wu, Q. and Jiang, S. (1994) Comparison of standard locked-ward treatment versus open-ward rehabilitation treatment for chronic schizophrenic patients: a one-year controlled trial in Canton. *The British Journal of Psychiatry*, 165 (S24): 45–51	China (Guangzhou- Canton)	Non-randomised control trial	1	90 (final measures on 86)	700	4.9–7.9 years	EPHPP (3) weak
9	RATS (score 7/25) weak	Fisher, F. D., Griffith, E. E. and Mahy, G. E. (1988) Recent developments in the Grenada mental health program. *Psychiatric Services*, 39 (9): 980–985	Grenada	Case study	1	–	150	–	RATS 7/25
10	RATS (score 5/25) weak	Ganesan, M. (2017) Transforming an out-of-date psychiatric hospital into a patient friendly space: a matter of taking risks. *Intervention*, 15 (1): 76–81	Sri Lanka (Colombo)	Personal reflection	1	–	900	–	RATS 5/25
11	EPHPP (score 2) moderate	Xiang, Y.-T., Weng, Y.-Z., Li, W.-Y., Gao, L., Chen, G.-L., Xie, L., Chang, Y.-L., Tang, W.-K. and Ungvari, G. S. (2007) Efficacy of the community re-entry module for patients with schizophrenia in Beijing, China: outcome at 2-year follow-up. *The British Journal of Psychiatry*, 190 (1): 49–56	China (Chaoyang Mental Health Care Institute)	Randomised control trial	1	103	4500 patients with schizophrenia receive OPD and IPD services	–	EPHPP (2) moderate
12	EPHPP (score 1) strong	Huf, G., Coutinho, E. and Adams, C. (2012) Physical restraints versus seclusion room for management of people with acute aggression or agitation due to psychotic illness (TREC-SAVE): a randomized trial. *Psychological Medicine*, 42 (11): 2265–2273	Brazil-Instituto philippe pinel, Rio de janeiro	Randomised control trial	1	105	70 in-patients and 30 emergencies per day	–	EPHPP (1) strong
13	Not rated	Hillman, A. (2007) Ruined lives: segregation from society in Argentina’s psychiatric asylums. *Washington DC: Mental Disability Rights International and Center for Legal and Social Studies*	Argentina- San Luis- Hospital Escuela de salud mental, San Luis- Cabred hospital San Luis-Borda Hospital	Report	Eight psychiatric hospitals across the country 3 (*reporting reform*)	–	Average of 1000 beds each	4–7 days Not mentioned for the other two hospitals	Not rated
14	Not rated	Murthy, P., Kumar, S., Desai, N. and Teja, B. (2015) Mental Health Care in India—old aspirations, renewed hope. *Report of the Technical Committee on Mental Health. New Delhi: National Human Rights Commission*	India	Report	47 hospitals in total Each hospital reported reform		79,947 in-patient admissions annually	Less than 1 month = 37% 1–3 months is 30% 3–6 months is 8% 6 months or more is 25%	Not rated
15	Not rated	Anon (2015) *Integrated community care for the needs of vulnerable people with severe Mental disorders, INCENSE; Grant completion report*. [online] Available from: http://hearingvoicescymru.org/wp-content/uploads/2016/03/Backup_of_INCENSE-Report-Final_11012016.pdf (Accessed May 13th)	India (*RMH *Pune, LGBRIMH-Assam)	Report	2	237 (200 in Pune and 37 in Tezpur)	–	Median duration of 12 years in Pune and 18 years in Tezpur	Not rated
16	Not rated	Nagaraja, D. and Murthy, P. (2008) Mental health care and human rights. *New Delhi: National Human Rights Commission*	India	Report	36		3,62,793 new registrations	–	Not rated

The qualitative synthesis enabled a more nuanced understanding of reform processes and outcomes. The sections below elucidate key findings on triggers for reform, key elements of structural and process reform, staff enrichment and capacity building programmes, and outcomes of reform.

### Triggers for reform

12 studies described a trigger for reform in mental hospital settings [19, 21, 23, 24, 27–32, 34, 36]. The reasons that triggered reform are listed in Table 4 and include poor quality of care particularly for long-stay patients in mental hospitals. India and Argentina report judicial intervention as a key trigger for reform in colonial era mental hospitals.

**Table 4:** Triggers for reform

Triggers of reform	No and % of studies	Country	References
Country level transformation of mental health care	2 (12.5%)	Brazil Grenada	[32, 37]
Judicial intervention	4 (25%)	India, Argentina	[19, 21, 23, 31]
Patients with long duration of hospital stay	2 (12.5%)	South Africa	[27, 28]
Suicide	1 (6.25%)	China	[24]
Poor quality of life for patients	3 (18.75%)	China, Sri Lanka	[24, 34, 36]
Need for evidence-based use of restraint or seclusion	1 (6.25%)	Brazil	[30]

### Elements of reform

Elements of reform described in the studies were categorised into structural reform (refurbishment of hospital infrastructure), process reform (reform of clinical and non-clinical hospital processes) and capacity building/training of hospital staff. Seven (43.75%) of the 16 studies included in the review reported structural elements of reform, captured in Table 5. In India, hospital infrastructure was improved [19, 21, 23], and community housing services established [20, 22]. In Georgia, a large 250-bedded hospital was closed and, in its place, multiple smaller 40-bedded units were established for long-stay patients [33]. In Argentina, hospital infrastructure was used to initiate half-way homes [31].

**Table 5:** Elements of structural reform

Elements of structural reform	Number and % of studies	Country	References
Closure of a large hospital and opening of smaller facilities	1 (6.25%)	Georgia	[33]
Infrastructural improvement	3 (18.75%)	India	[19, 21, 23]
Half way home within the hospital	1 (6.25%)	Argentina	[31]
Community living services	2 (12.5%)	India	[20, 22]

Process reform was reported in 14 (87.5%) studies. This has been categorised and reported in Table 6. We have discussed these reforms by each country in this section to provide a comprehensive picture.

**Table 6:** Elements of process reform

Elements of process reform	No and % of studies	Country	References
Reform in the process of medical management	4 (25%)	India, South Africa, Grenada, and Argentina	[21, 28, 31, 32]
Reform in admission process from custodial to voluntary	2 (12.5%)	India	[21, 23]
Introduction of open wards	6 (37.5%)	India, China, and Argentina	[21–24, 31, 36]
Introduction of community-based services linked to the hospital	3 (18.75%)	India, China	[22, 23, 26]
Reform of restraint and seclusion procedures	4 (25%)	India, Brazil, Grenada, and Sri Lanka	[23, 30, 32, 34]
Introduction of mixed gender wards to promote social interaction	1 (6.25%)	Brazil	[29]
Patient involvement in hospital management	1 (6.25%)	China	[24]
Promotion of an equal relationship between staff and patients	1 (6.25%)	China	[24]
Structured discharge planning	3 (18.75%)	China, Grenada, and South Africa	[26, 27, 32]
Change in nursing practice to increase patient interaction	1 (6.25%)	Sri Lanka	[34]
Introduction of psychosocial interventions including ADL, employment and other rehabilitation activities	5 (31.25%)	India, China	[20, 22, 24, 26, 36]
Engagement with family	2 (12.5%)	India, China	[21, 26]
Introduction of colored clothes instead of uniforms for patients	1 (6.25%)	China	[24]
Introduction of a meal management system (buffet)	1 (6.25%)	Sri Lanka	[34]

There were several process reforms initiated in Indian mental hospitals, including reforms in medical management [21], There was a shift in process of admissions with hospitals moving away from admissions through a legal intervention to voluntary admissions [21, 23]. There was an initiation of more open wards as evidenced in two country-level reports, shift in restraint and seclusion processes, and a reduction in use off custodial cells for isolation [21, 23]. Simultaneously, community-based services linked to mental hospitals were also initiated [23]. An introduction of psychosocial interventions along with a focus on Activities of Daily Living (ADL) and rehabilitation services including employment was reported [20, 22] along with greater inclusion and involvement of family members in the treatment and care process [21].

In China, process reforms in mental hospitals saw a move from closed to open wards [25], and initiation of community-based services in tandem with mental hospitals [26]. Further, patient involvement in hospital management and promotion of non-hierarchical relationships between staff and patients through a structured engagement process was reported [24]. Psychosocial interventions were introduced [24–26] along with discharge planning, structured community re-entry and the involvement of family [26]. Other reforms included introduction of personal/coloured clothes instead of uniforms for patients living in mental hospitals [24].

Argentina shifted towards open wards and reform in medical management of patients in hospital [31] while reforms in Brazil included changes in restraint and seclusion practices [30] and the introduction of mixed gender wards to promote social interaction amongst patients [37]. Process reforms in South Africa composed of changes in medical management and clinical services [28] along with structured discharge planning [27]. Grenada’s process reforms composed of changes in medical management and structured discharge planning [32].

Sri Lanka saw a change in restraint and seclusion practices, change in nursing practices (involvement of nursing staff in intake assessment and treatment planning) and changes in the way meals were distributed to patients with the introduction of a buffet-style self-service system [34].

### Capacity building

Six (37.5%) of 16 studies reported capacity building of staff and covered four countries (Table 7). Mental hospitals in India are being reformed as teaching and research institutions with the introduction of formal teaching programs [19, 21, 23]. Grenada reported a formal training programme for staff along with recreational activities, such as a multi-disciplinary journal club, to augment staff capabilities [32]. Mental hospital reforms in Sri Lanka included staff engagement as a means of bringing change in practices [34] and South Africa reported a trained team dedicated to the care of long-stay patients [28].

**Table 7:** Hospital staff training and capacity building

Elements of staff training and capacity building	No and % of studies	Country	References
Development of mental hospitals as teaching and research institutes	3 (18.75%)	India	[19, 21, 23]
Formal training programme for hospital staff	1 (6.25%)	Grenada	[32]
Staff engagement in changing of practice	1 (6.25%)	Sri Lanka	[34]
Trained and dedicated team for management of long-stay patients	1 (6.25%)	South Africa	[28]

### Outcomes of reform

Outcome measures were reported by seven (43.75%) of 16 studies from South Africa, India, China, Brazil and Grenada [22, 24, 26, 27, 30, 32, 36] (Table 8).

**Table 8:** Outcomes of reform

Type of outcome	Outcomes	No and % of studies	Country	References
Clinical	Improvement in psychiatric symptoms	2 (12.5%)	China	[26, 36]
	Reduction in relapse of illness	2 (12.5%)	China	[24, 26]
	Reduction in time in restraint/isolation	1 (6.25%)	Brazil	[30]
	Reduction in suicide	1 (6.25%)	China	[24]
	Discharge of patients from hospital	2 (12.5%)	Grenada, Brazil	[30, 32]
Functional	Improvement in personal appearance	1 (6.25%)	China	[36]
	Improvement in engagement with employment	2 (12.5%)	China	[24, 26]
Social	Improvement in staff and patient interaction	2 (12.5%)	South Africa, China	[24, 27]
	Improvement in interactions with family/integration with family	2 (12.5%)	China, India	[22, 24]
	Improvement in overall social functioning	2 (12.5%)	China	[26, 36]

In China, clinical outcomes reported improvement in psychiatric symptoms [25, 26], reduction in episodes of relapse [24, 26] along with a reduction in suicides [24]. Functional outcomes reported were an improvement in personal appearance [25] and improvement in engagement with employment [24, 26]. Social outcomes of reform reported were improvement in staff and patient interactions which was also reported from South Africa [24, 27], improvement in interaction with family [24] and improvement in overall social functioning [25, 26]. Brazil reported a reduction of time in restraints through the use of seclusion as a technique instead of mechanical restraints [30] and discharge of patients from the hospital [30]. Grenada saw the discharge of patients from hospital as a clinical outcome of reform emphasising short-term care and rapid return of patients to the community. [32]. India reported integration with family as a social outcome of reform [22].

It was particularly interesting to note that none of the studies report data on costs incurred for reform.

## Discussion

This review was undertaken with an objective to bring together research on psychiatric hospital reform in LMICs to understand the process of reform and patient-related outcomes as a result of reform. The review aimed to identify gaps in current evidence and research with regard to the reform of psychiatric institutions in LMICs.

The conceptual framework used for this narrative review was based on the review question and explored the relationship between the circumstances that propelled change or reform in mental hospitals and the elements of reform and patient outcomes associated with the reform.
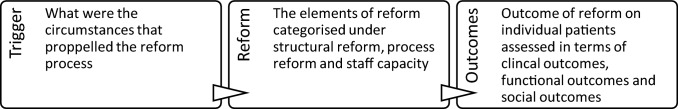


### Dearth of research

There is clearly a dearth of research on mental hospital reform processes. We found only 16 studies from 137 countries across a period of four decades. State-run mental hospitals continue to play a key role in providing services in most parts of the world. They deal with an increasingly challenging population with a large number of people having extended periods of hospital stay [1, 3]. Downsizing of mental hospitals and deinstitutionalization comes ridden with its own problems of trans-institutionalization, homelessness and imprisonment of people living with severe mental illness [38–41]. In such a scenario, mental hospitals need to reinvent themselves to meet the needs of the very vulnerable population they serve. Psychiatric hospital reform needs to be backed by robust evidence on the process of reform and its clinical, social and functional outcomes and the costs thereof. This is a key requirement for governments and policy-makers to make informed decisions and improve the landscape of mental health service delivery.

### Drivers of reform

Change or reform appears to be driven by the need to make a difference in the quality of life of long-stay patients [24, 25, 27, 28, 34]. Often such reform is catalysed by judicial action or higher-level reform of the country’s mental health system as seen in India, Argentina and Brazil [19, 21, 23, 31, 32, 37] The need for improved clinical practice and reduction in violation of basic human rights also triggered reform as evidenced by the modified use of restraints or seclusion as in the case of Brazil [30, 35].

### Optimisation of resources

Mental hospitals in most parts of the world have been established during the colonial era and urgently require refurbishment or renovation of the old infrastructure. As hospitals were downsized, their infrastructure was modified to create facilities that more appropriately serve patient needs. For instance, using old hospital wards as a half-way-home facility as seen in Argentina [31] and the creation of open wards as in China and Brazil [25, 37]. In India, several infrastructural changes have been carried out across hospitals to improve living conditions for patients [19, 21, 23]. Infrastructural changes have also been associated with the simultaneous development of community living services [20, 22] while downsizing hospitals into more compact acute care units [33].

### Process reform as a catalyst to improve quality of life

Although often unplanned, most reforms seem to be centered around a change in processes. Reform of processes—largely comprising shifts in clinical and medical management protocols is directly linked to improving the quality of life for patients in mental hospitals [21, 23, 26–28, 30–32, 34, 35]. Large-scale shifts, such as changes in admission processes and moving from custodial to voluntary admissions [21, 23], the introduction of open wards [21–25, 31], greater integration of psychosocial services and an incremental push towards improving autonomy and dignity of long-stay patients, have been seen globally [20, 22, 24–26, 34, 37]. Further, greater attention has been paid towards more intangible, and experiential elements of care. Shifts in clothing policies, food service timings and processes, and access to leisure and recreation have all contributed significantly to an improvement in functioning and overall quality of life.

### Mental health professionals as key drivers of recovery-oriented practice

Capacity building of staff was seen as an associated and significant piece of the reform process where countries like India [19, 21, 23] have made a central push for all mental hospitals to transform into centres of excellence that are front runners of training, research and knowledge creation. Training of staff has been, in varying degrees, an important conduit of reform in psychiatric hospital settings [28, 32, 34].

### Study limitations

A major limitation of this review is the variable quality of the studies included with most studies being of poor quality. Further, published literatures from countries that have experienced massive mental health reform, such as that of Brazil [42], are available in languages other than English. Their inclusion was beyond the scope of this review as a result of limited resources available. This also limited our ability to include grey literature in the scope of our review. Detailed quantitative analysis is limited by the quality of included studies as well as the variability in measures. This has implications on the extent of evidence and its ability to answer the question this review focused on which is the extent of scientific evidence around psychiatric hospital reform and its associated patient-related outcomes in the context of low- and middle-income countries.

## Conclusion

Mental hospitals remain an integral part of psychiatric services globally. In some parts of the world, these hospitals form a majority of, and in some cases, the entire service continuum [43]. Mental hospitals however are not static entities, but are evolving and finding renewed relevance in the global landscape of de-institutionalization and community-based services [2]. Currently, reform of hospitals appears unplanned and de-linked to evidence. Reforms do not appear to be linked to patient outcomes. There is a large gap in scientific evidence that needs to be bridged urgently such that future reform processes may be more informative. Further research could investigate the correlational and causal pathways between reform and patient outcomes, clearly determine the costs of the reforms, and discern whether they require a radical shift in human resource allocations. In addition, we also believe social scientists (psychologists, anthropologists, sociologists, economists, etc.) could study the impact of culture, social norms and value systems on mental health service provision in LMICs.

## Data Availability

Not applicable.

## References

[1] WHO. Mental health atlas 2017. Geneva: World Health Organization; 2018. World Health Organization WHO MiNDbank. http://www.who.int/mental_health/mindbank/en. Accessed 1 Sept 2020

[2] Chatterjee S (2017). Time to focus on institutional reforms in low and middle income countries. Epidemiol Psychiatr Sci.

[3] Fisher WH, Geller JL, Pandiani JA (2009). The changing role of the state psychiatric hospital. Health Aff.

[4] Cohen A, Minas H (2017). Global mental health and psychiatric institutions in the 21st century. Epidemiol Psychiatr Sci.

[5] Dowdall GW (1999). Mental hospitals and deinstitutionalization. Handbook of the sociology of mental health.

[6] Frankish H, Boyce N, Horton R (2018). Mental health for all: a global goal. Lancet.

[7] Patel V, Xiao S, Chen H (2016). The magnitude of and health system responses to the mental health treatment gap in adults in India and China. Lancet.

[8] Collins PY, Patel V, Joestl SS, March D, Insel TR, Daar AS (2011). Grand challenges in global mental health. Nature.

[9] Chatterjee S, Patel V, Chatterjee A, Weiss HA (2003). Evaluation of a community-based rehabilitation model for chronic schizophrenia in rural India. Br J Psychiatry.

[10] Kleinman A (2009). Global mental health: a failure of humanity. The Lancet.

[11] Kohn R, Saxena S, Levav I, Saraceno B (2004). The treatment gap in mental health care. Bull World Health Organ.

[12] Patel V (2007). Mental health in low-and middle-income countries. Br Med Bull.

[13] Saxena S, Thornicroft G, Knapp M, Whiteford H (2007). Resources for mental health: scarcity, inequity, and inefficiency. Lancet.

[14] Moher D, Altman DG, Liberati A, Tetzlaff J (2011). PRISMA statement. Epidemiology.

[15] Raja T, Tuomainen H, Madan J, Mistry D, Jain S, Singh S (2020). Psychiatric hospital reform in low-income and middle-income countries structured individualised intervention and recovery (SITAR): a two-arm pragmatic randomised controlled trial study protocol. BMJ Open.

[16] Godlee F, Jefferson T, Callaham M (2003). Peer review in health sciences.

[17] Leamy M, Bird V, Le Boutillier C, Williams J, Slade M (2011). Conceptual framework for personal recovery in mental health: systematic review and narrative synthesis. Br J Psychiatry.

[18] Evans N, Lasen M, Tsey K (2015). Appendix A: Effective public health practice project (EPHPP) quality assessment tool for quantitative studies. A systematic review of rural development research briefs in public health.

[19] Cochrane: data collection form for intervention reviews: RCTs and non-RCTs version 3, April 2014. https://dplp.cochrane.org/data-extraction-forms. Accessed 20 Feb 2020

[20] Varma S (2016). Disappearing the asylum: modernizing psychiatry and generating manpower in India. Transcult Psychiatry.

[21] Murthy P, Isaac M, Dabholkar H (2017). Mental hospitals in India in the 21st century: transformation and relevance. Epidemiol Psychiatr Sci.

[22] Murthy P, Kumar S, Desai N, Teja B (2016). Mental health care in India—old aspirations, renewed hope. Report of the technical committee on mental health.

[23] Integrated community care for the needs of vulnerable people with severe mental disorders (2015) INCENSE; Grant completion report. http://hearingvoicescymru.org/wp-content/uploads/2016/03/Backup_of_INCENSE-Report-Final_11012016.pdf. Accessed 13 May 2018

[24] Nagaraja D, Murthy P (2008). Mental health care and human rights.

[25] Jin D, Li G (1994). The role of human rights and personal dignity in the rehabilitation of chronic psychiatric patients. A rural therapeutic community in Yanbian, Jilin. Br J Psychiatry Suppl.

[26] Fan Z, Huang J, Wu Q, Jiang S (1994). Comparison of standard locked-ward treatment versus open-ward rehabilitation treatment for chronic schizophrenia patients: a one-year controlled trial in Canton. Br J Psychiatry.

[27] Xiang Y-T, Weng Y-Z, Li W-Y, Gao L, Chen G-L, Xie L (2007). Efficacy of the community re-entry module for patients with schizophrenia in Beijing, China: outcome at 2-year follow-up. Br J Psychiatry.

[28] Uys LR, Mhlaluka NG, Piper SE (1996). An evaluation of the effect of programme changes in an acute psychiatric unit. Curationis.

[29] Kruger C, Lewis C (2011). Patient and social work factors related to successful placement of long-term psychiatric in-patients from a specialist psychiatric hospital in South Africa. Afr J Psychiatry.

[30] Bandeira PM, Souza CHP, da Silva Guimarães JC, de Almeida Filho AJ, de Almeida Peres MA (2015). Psychiatric nursing in integrated wards accommodating both female and male patients: a historic pioneering reform initiative implemented by the institute of psychiatry, a unit of the federal university of Rio De Janeiro, Brazil. Issues Ment Health Nurs.

[31] Huf G, Coutinho E, Adams C (2012). Physical restraints versus seclusion room for management of people with acute aggression or agitation due to psychotic illness (TREC-SAVE): a randomized trial. Psychol Med.

[32] Hillman A (2007). Ruined lives: segregation from society in Argentina’s psychiatric asylums.

[33] Fisher FD, Griffith EE, Mahy GE (1988). Recent developments in the Grenada mental health program. Hosp Community Psychiatry.

[34] Makhashvili N, van Voren R (2013). Balancing community and hospital care: a case study of reforming mental health services in Georgia. PLoS Med.

[35] Ganesan M (2017). Transforming an out-of-date psychiatric hospital into a patient friendly space: a matter of taking risks. Intervention.

[36] Huf G, Coutinho ES, Ferreira MA, Ferreira S, Mello F, Adams CE (2011). TREC-SAVE: a randomised trial comparing mechanical restraints with use of seclusion for aggressive or violent seriously mentally ill people: study protocol for a randomised controlled trial. Trials.

[37] Fan Z, Huang J, Wu Q, Jiang S (1994). Comparison of standard locked-ward treatment versus open-ward rehabilitation treatment for chronic schizophrenic patients: a one-year controlled trial in Canton. Br J Psychiatry.

[38] Bandeira PM, Haddad PSC, da Silva Guimaraes JC, de Almeida Filho AJ, de Almeida Peres MA (2015). Psychiatric nursing in integrated wards accommodating both female and male patients: a historic pioneering reform initiative implemented by the institute of psychiatry, a unit of the federal university of Rio De Janeiro, Brazil. Issues Ment Health Nurs.

[39] Lamb HR, Weinberger LE (2001). Persons with severe mental illness in jails and prisons: a review. New Dir Stud Leadersh.

[40] Thornicroft G, Bebbington P (1989). Deinstitutionalisation–from hospital closure to service development. Br J Psychiatry.

[41] Carr S (2018). Implementing sustainable global mental health in a fragmenting world. Lancet.

[42] The LP (2015). The land of the free. Lancet Psychiatr.

[43] Candiago RH, Saraiva Sda S, Goncalves V, Belmonte-de-Abreu P (2011). Shortage and underutilization of psychiatric beds in southern Brazil: independent data of Brazilian mental health reform. Soc Psychiatry Psychiatr Epidemiol.

